# JAC4 Alleviates Rotenone-Induced Parkinson’s Disease through the Inactivation of the NLRP3 Signal Pathway

**DOI:** 10.3390/antiox12051134

**Published:** 2023-05-20

**Authors:** Lu Zou, Zhen Che, Kun Ding, Chao Zhang, Xia Liu, Luman Wang, Aiping Li, Jianwei Zhou

**Affiliations:** 1 Department of Molecular Cell Biology & Toxicology, Center for Global Health, School of Public Health, Nanjing Medical University, Nanjing 211166, China; 2 The Key Laboratory of Modern Toxicology of Ministry of Education, School of Public Health, Nanjing Medical University, Nanjing 211166, China

**Keywords:** Parkinson’s disease, rotenone, JAC4, mitochondrial complex I, oxidative stress, NLRP3 inflammasome

## Abstract

Parkinson’s disease (PD) is the fastest-growing neurodegeneration disease, characterized typically by a progressive loss of dopaminergic neurons in the substantia nigra, and there are no effective therapeutic agents to cure PD. Rotenone (Rot) is a common and widely used pesticide which can directly inhibit mitochondrial complex I, leading to a loss of dopaminergic neurons. Our previous studies proved that the JWA gene (arl6ip5) may play a prominent role in resisting aging, oxidative stress and inflammation, and JWA knockout in astrocytes increases the susceptibility of mice to 1-Methyl-4-phenyl-1,2,3,6-tetrahydropyridine (MPTP)-induced PD. JWA-activating compound 4 (JAC4) is a small-molecule activator of the JWA gene, but its role in and mechanism against PD have not yet been clarified. In the present study, we showed that the JWA expression level is strongly related to tyrosine hydroxylase (TH) in different growth periods of mice. Additionally, we constructed models with Rot in vivo and in vitro to observe the neuroprotective effects of JAC4. Our results demonstrated that JAC4 prophylactic intervention improved motor dysfunction and dopaminergic neuron loss in mice. Mechanistically, JAC4 reduced oxidative stress damage by reversing mitochondrial complex I damage, reducing nuclear factor kappa-B (NF-κB) translocation and repressing nucleotide-binding domain, leucine-rich-containing family and pyrin domain-containing-3 (NLRP3) inflammasome activation. Overall, our results provide proof that JAC4 could serve as a novel effective agent for PD prevention.

## 1. Introduction

Parkinson’s disease (PD) is the second most common neurodegenerative disease associated with aging, characterized by a loss of dopaminergic neurons and the formation of Lewy bodies in the substantia nigra [1,2]. The incidence of PD increases dramatically with age, and the number of people with PD will exceed 12.9 million by 2040 due to the world’s aging population [3]. However, there is currently no treatment that can cure or slow the progression of PD. Therefore, the development of effective agents to prevent or treat PD is an urgent and unmet need worldwide.

The etiology of PD remains largely unknown. Aging is probably the most important risk factor for PD, which is also related to genetic and environmental factors [4]. Pesticide exposure is one of the most important environmental risk factors [5]. Mitochondria and aging are closely related to PD [6]. Rotenone (Rot) is a common and widely used pesticide, which can directly inhibit mitochondrial complex I and induce dopaminergic neuron loss in the substantia nigra, in addition to triggering Lewy body formation [7,8]. Moreover, the inhibition of complex I activity has been observed in the autopsy brain tissues of PD patients [9]. Epidemiological studies have shown a 2.5-5.8-times increased incidence of PD in people with long-term Rot-exposure [10]. The NLRP3 inflammasome has been found in the blood of PD patients, indicating that the NLRP3 inflammasome may be involved in the pathogenesis of PD [11]. Additionally, the activation of NLRP3 usually requires the involvement of reactive oxygen species (ROS) and NF-κB [12].

The JWA gene [13], also known as ADP ribosylation factor-like GTPase 6 interacting protein 5 (arl6ip5) (GenBank: AF070523, 1998), is an active environmental response gene involved in responses to oxidative stress and inflammation [14], the repair of DNA damage [15] and anti-aging [16]. It has been found that astrocyte JWA knockout aggravates motor dysfunction, dopaminergic neuron loss and astrocyte activation in PD mice [17]. It has also been demonstrated that neurons can be protected through the JWA-IKKβ-NF-κB signaling pathway [18]. However, the specific regulatory mechanism between JWA and inflammasome has not been elucidated.

JAC4 is a small-molecule compound activating the JWA gene that was screened in a series of high-throughput screening assays conducted by the Chinese National Compound Library [14,19]. In this study, we demonstrated, for the first time, that JAC4 can alleviate oxidative stress and inflammation, which antagonizes Rot-induced dopaminergic neuronal degeneration. Further exploration of the mechanism showed that JWA suppresses the activation of the NLRP3 inflammasome partially through the AKT/GSK3β/NF-κB signaling pathway. Our study may provide a new target and therapeutic strategy for PD.

## 2. Materials and Methods

### 2.1. Animal and Experimental Design

The animal experiment was approved by the Ethics Committee of Nanjing Medical University (IACUC-2004044). For the model of mice in different growth stages, all male C57BL/6 mice were bred in our laboratory and born in the same week. These mice were randomly divided into four groups (*n* = 3) and terminated at different ages. For the Rot-induced PD model, male C57BL/6 mice (7 weeks old, 23–25 g) were provided by the SLAC Laboratory Animal Center (Shanghai, China). Rot was purchased from Sigma-Aldrich Chemical Co. JAC4 (C_16_H_13_N_3_O_3_S, MW: 327.4) was synthesized in our laboratory. The chemical structure formula of JAC4 is shown in Figure S1A. The solvent composition contained 52.5% saline, 40% polyethylene glycol, and 7.5% absolute ethanol. The mice were randomly divided into four groups (*n* = 10): a normal control group, disease model group, JAC4 prophylactic administration group and JAC4 therapeutic administration group. The mice in the JAC4 prophylactic administration group were given JAC4 (100 mg/kg body weight/day) via gavage for three days before Rot administration, and the mice in the other groups were given equal volumes of solvent. Except for the normal control group, each group was orally given Rot (30 mg/kg body weight/day) for 46 days and treated with JAC4 or solvent. The mice in the JAC4 therapeutic administration group were given JAC4 treatment after behavioral dysfunction was observed. Body weight was measured every four days. Behavioral tests were performed within the last week before the termination of the animal model.

### 2.2. Behavioral Test

The open field test was used to test spontaneous movement ability. The mice were acclimated in the chamber for 20 min before the test began. The overall distance (mm) and track were recorded in 5 min using Open Field software (Clever Sys Inc., Reston, VA, USA).

The pole test is performed to observe bradykinesia. The mice were placed on the top of the pole, and the time that mice took to climb down to the floor was recorded. The pole was 1 cm in diameter and 50 cm in height. The test was carried out 3 times, and the average time was calculated.

The rotarod test was used to evaluate motor coordination. The mice were trained 3 times on an accelerating rotarod before the test. In every training session or test, the rotarod started at 0 rpm, accelerated to 20 rpm within 1 min and then maintained a constant speed of 20 rpm. The latency time taken to fall from the rotarod was recorded during the 5 min test. 

### 2.3. Cell Culture and Transfection

HT-22 and SH-SY5Y cells were cultured in Dulbecco’s Modified Eagle’s Medium supplemented with 10% fetal bovine serum (FBS; Gibco, Grand Island, NY, USA) and 1% penicillin–streptomycin (HyClone, Logan, UT, USA). All the cells were incubated at 37 °C in a humidified incubator containing 5% CO_2_. For cell transfection, the cells were transfected with shNC and shJWA plasmids for 48 h using lipo 8000 reagent (Beyotime, Shanghai, China). The shJWA plasmid’s construction was conducted with reference to the previous research conducted in our laboratory [16].

### 2.4. Cell Viability Assay

The cells were seeded in 96-well plates 24 h before treatment (5 × 10^3^ cells per well). In the cytotoxicity test using Rot, cells were treated with Rot at different doses for 24 h to detect its toxicity. In the test for JAC4’s effect on Rot-induced injury, cells were pretreated with JAC4 for 24 h and then co-treated with Rot for 48 h. In addition, cells were treated with JAC4 alone for 72 h to observe the cytotoxicity of JAC4. Next, 10% CCK 8 solution (100 μL) was added into each well for 2 h of incubation after treatment. The absorbance was measured at 450 nm. Five biological replications were performed. 

### 2.5. Western Blot Assay

Cells or mice brain tissue samples were lysed with RIPA buffer containing phosphatase inhibitor and protease inhibitor for 30 min at 4 °C. The protein concentration was measured using a BCA Protein Assay Kit (Beyotime, Shanghai, China). The same amount of protein (20 μg/10 μL) was separated via SDS-PAGE and transferred to the PVDF membrane. The membranes were incubated with the primary antibodies overnight at 4 °C after 5% defatted milk blocking for one hour at room temperature. Additionally, they were then incubated with the corresponding peroxidase-conjugated anti-rabbit IgG or anti-mouse IgG for 1 h after washing for 10 min (3×) with TBST. Subsequently, the blot images were obtained using the Tanon-5200Multi Fluorescence Image System and analyzed using Image J. The antibodies used in this experiment are as follows: anti-JWA (1:100, AbMax, Beijing, China), anti-caspase-3 (1:1000, Cell Signaling Technology, Danvers, MA, USA), anti-caspase-9 (1:1000, Cell Signaling Technology, Danvers, MA, USA), anti-cleaved-PARP1 (1:1000, Cell Signaling Technology, Danvers, MA, USA), anti-PARP1 (1:1000, Servicebio, Wuhan, China), anti-TH (1:1000, Cell Signaling Technology, Danvers, MA, USA), anti-NLRP3 (1:1000, Servicebio, Wuhan, China), anti-MT-ND1 (1:1000, Cell Signaling Technology, Danvers, MA, USA), anti-AKT (1:1000, Abcam, Cambridge, UK), anti-p-AKT (1:1000, Abcam, Cambridge, UK), anti-GSK3β (1:1000, Cell Signaling Technology, Danvers, MA, USA), anti-p-GSK3β (Ser9) (1:1000, Cell Signaling Technology, Danvers, MA, USA), anti-p-GSK3β (Tyr216) (1:1000, Beyotime, Shanghai, China), anti-NF-κB (1:1000, Cell Signaling Technology, Danvers, MA, USA), anti-Lamin B1 (1:10,000, Abcam, Cambridge, UK), anti-α-synuclein (1:5000, Abcam, Cambridge, UK), anti-p-α-synuclein (Ser 129) (1:1000, Abcam, Cambridge, UK), anti-dopamine transporter (1:1000, Abcam, Cambridge, UK), anti-β-actin (1:1000, Beyotime, Shanghai, China), anti-GAPDH (1:1000, Beyotime, Shanghai, China) anti-Tubulin (1:1000, Beyotime, Shanghai, China), HRP-labeled Goat Anti-Rabbit IgG(H + L) (1:1000, Beyotime, Shanghai, China) and HRP-labeled Goat Anti-Mouse IgG(H + L) (1:1000, Beyotime, Shanghai, China).

### 2.6. Quantitative Real-Time PCR Analysis

Total RNA was extracted using Trizol reagent and reversed-transcribed into cDNA. qRT-PCR was performed in a reaction system containing cDNA, primers and SYBR (TaKaRa Bio, Kusatsu, Japan) with an ABI system (Applied Biosystems, Mississauga, ON, Canada). GAPDH was used as a control for every sample. The primer sequences are as follows: JWA (BC003897), forward 5′-CGGCATCACTCTTCCTTTGCTG-3′ (Tm: 59.04) and reverse 5′-CTTCCTGCTGTTCCAAGGCATC-3′ (Tm: 58.01); NLRP3 (BC116174), forward 5′-TCACAACTCGCCCAAGGAGGAA-3′ (Tm: 59.87) and reverse 5′-AAGAGACCACGGCAGAAGCTAG-3′ (Tm: 58.14); Caspase-1 (BC008152), forward 5′-GGCACATTTCCAGGACTGACTG-3′ (Tm: 57.57) and reverse 5′-GCAAGACGTGTACGAGTGGTTG-3′ (Tm: 58.21); IL-1β (BC011437), forward 5′-TGGACCTTCCAGGATGAGGACA-3′ (Tm: 56.87) and reverse GTTCATCTCGGAGCCTGTAGTG-3′ (Tm: 56.31); IL-18 (BC024384), forward 5′-GACAGCCTGTGTTCGAGGATATG-3′ (Tm: 57.41) and reverse TGTTCTTACAGGAGAGGGTAGAC-3′ (Tm: 54.93); NDUFS2 (BC016097), forward 5′-CGTTTACGACCAGGTGGAGTTTG (Tm: 58.28) and reverse GAGGCATCTTGTTCAGACACTGC-3′ (Tm: 58.32); and GAPDH (BC083065), forward 5′-GCCGGTGCTGAGTATGTC-3′ (Tm: 59.59) and reverse 5′-CTTCTGGGTGGCAGTGAT-3′ (Tm: 61.43).

### 2.7. Biochemical Analysis

Mice plasma and midbrain tissues were strictly detected according to the protocol for the malondialdehyde (MDA), superoxide dismutase (SOD) and glutathione (GSH) kit (Beyotime, Nanjing, China). Three biological replications were performed for all the experiments.

### 2.8. Intracellular ROS Assay

After the JAC4 and Rot treatments, the cells were incubated with DCFH-DA (concentration: 10 μM, Beyotime, Shanghai, China) for 30 min at 37 °C to measure the level of ROS, and the fluorescence intensity of the cells was observed using an inverted fluorescence microscope (Nikon, Tokyo, Japan). Three biological replications were performed.

### 2.9. Measurement of Mitochondrial Membrane Potential

The mitochondrial membrane potential (MMP, ΔΨm) was measured using a JC-1 probe (Beyotime, Shanghai, China). The cells were incubated with JC-1 for 30 min at 37 °C and then washed 2 times with JC-1 buffer. When ΔΨm decreased, the red fluorescence of the mitochondria decreased and the green fluorescence increased. The results were observed using an inverted fluorescence microscope and analyzed using the red/green ratio. Three biological replications were performed.

### 2.10. Measurement of ATP

After the JAC4 and Rot treatments, we added cell lysis buffer for 30 min at 4 °C. Then, cell lysates were collected and centrifugated at 12,000× *g* for 5 min. The content of ATP in the cells were measured using the ATP Assay Kit (S0026, Beyotime, Shanghai, China) according to the protocol and detected using a luminometer. Meanwhile, the results were normalized using the protein concentration and presented in nmol/mg protein. Three biological replications were performed.

### 2.11. Immunofluorescence Assay

The cells were seeded into confocal dishes at a density of approximately 5%. They were then fixed with methanol for 30 min after treatment and further supplemented with normal sheep serum in a 1:100 concentration to block the non-specific binding sites for 1 h at room temperature. Then, the cells were incubated with primary antibodies overnight at 4 °C and incubated with a secondary antibody at room temperature in the dark for 1 h. Finally, the cells were supplemented with Antifade Mounting Medium with DAPI for 15 min in the dark and observed with a laser-scanning Zeiss LSM 700 confocal microscope system (Carl Zeiss Jena, Oberkochen, Germany). For the immunofluorescence assay of the frozen sections of brain tissues, we referred to the steps above. The antibodies used in this experiment are as follows: anti-NF-κB (1:500, Cell Signaling Technology, Danvers, MA, USA), TH (1:200, Cell Signaling Technology, Danvers, MA, USA), GFAP (1:200, Cell Signaling Technology, Danvers, MA, USA), IBA-1 (1:100, Abcam, Cambridge, UK), Goat Anti-Mouse IgG H & L (Alexa Fluor^®^ 555) (1:500, Abcam, Cambridge, UK) and Goat Anti-Rabbit IgG H&L (Alexa Fluor^®^ 488) (1:500, Abcam, Cambridge, UK). The positive cell count was determined using Image J 1.8.0 software after selecting the SNc according to the stereotaxic coordinates of the mouse brain by manual means (*n* = 3).

### 2.12. Statistics Analysis

SPSS 22.0 and GraphPad Prism 8.0 were used for the statistical analysis. For gene differential expression analysis using the GEO database, relevant gene data were extracted and examined with the Log_2_(*n*) change to analyze the differences between samples. The data were calculated as the mean ± S.E.M and assessed with the unpaired Student’s *t*-test or ANOVA followed by Tukey’s test. Additionally, the correlation between the two variables was analyzed using Pearson’s correlation. *p* < 0.05 was considered to mark a statistical difference.

## 3. Results

### 3.1. The Expression of JWA Positively Correlates with TH in PD

**Figure 1:**
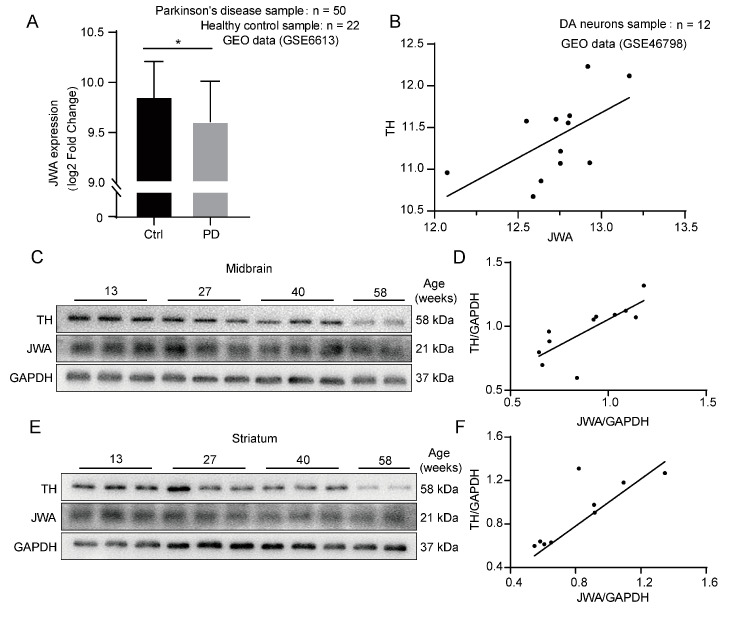
The expressions of JWA and TH are positively correlated in human and mice. (**A**) JWA mRNA expression was measured in a case–control study (GSE6613, 50 PD samples and 22 healthy samples). (**B**) Correlation analysis of the expressions of JWA and TH in DA neurons. (GSE 46798, *n* = 12, r =0.59). (**C**–**F**) The protein expressions of TH and JWA in midbrain (**C**) and striatum (**E**) of mice in different growth stages and the correlation analysis of the quantitative results (r = 0.73 in (**D**), r = 0.77 in (**F**)). (* *p* < 0.05).

To determine the role of JWA in the occurrence and development of PD, we analyzed the microarray data from the GEO database and found that the mRNA expression of JWA in whole blood from the PD samples (*n* = 50) was significantly lower than that of the healthy samples (*n* = 22, Figure 1A). Furthermore, Pearson’s correlation analysis showed that there was a positive correlation between TH and JWA expression levels in dopamine neurons (*n* = 12, Figure 1B). We also measured the expressions of TH and JWA in the midbrain and striatum of mice of different ages. Western blot analysis showed that the expressions of both TH and JWA decreased gradually with age and presented the same trend (Figure 1C–F). These data suggest that JWA may serve as an important regulator of PD progression.

### 3.2. JAC4 Reduces Dopaminergic Neuron Loss and NLRP3 Inflammasome Activation in Rot-Induced PD Mice

**Figure 2:**
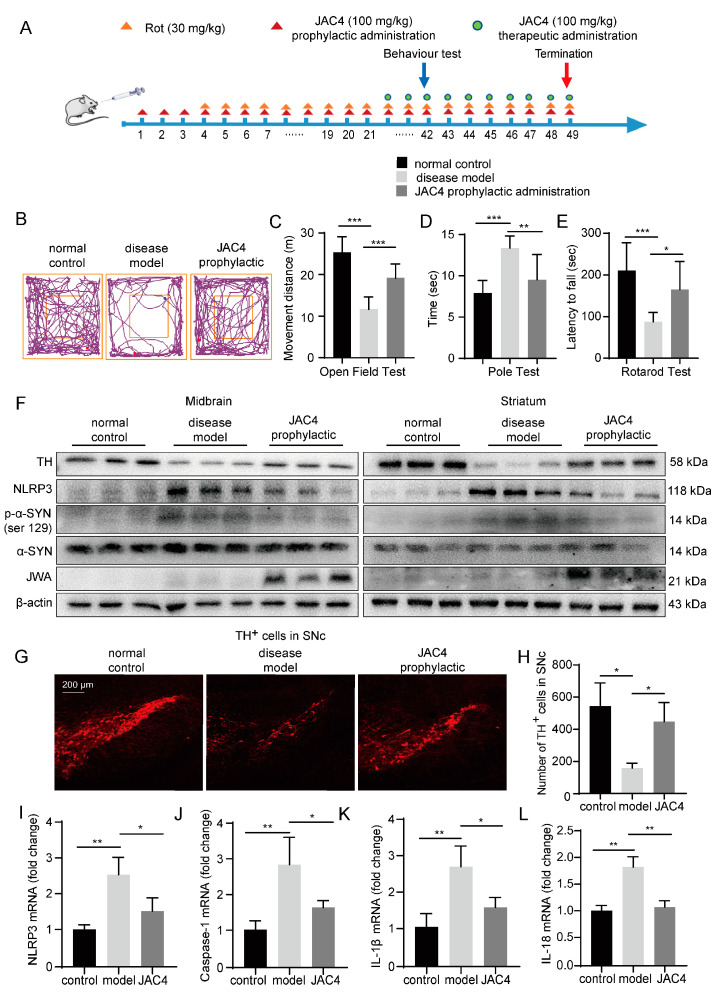
JAC4 reduces dopaminergic neuron loss and NLRP3 inflammasome activation in rotenone-induced PD mice. (**A**) Schematic diagram of the experimental design of the mice (*n* = 10 mice/group). (**B**–**E**) Behavior test results for the normal control, disease model and JAC4 prophylactic administration mice. Movement track (**B**) and distance (**C**) of mice over 5 min in the open field. Climbing time from the top to the bottom of the pole in the pole test (**D**). Latency to fall within 5 min in the rotarod test (**E**). (**F**) The protein expression levels of TH, NLRP3, α-SYN, p-α-SYN (Ser129) and JWA in the midbrain and striatum. (**G**,**H**) Immunofluorescence image and quantification of TH-positive neurons in SNc (*n* = 3). (**I**–**L**) The mRNA expression of NLRP3, caspase-1, IL-1β and IL-18 in the midbrain. The results are shown as the mean ± SEM (*n* = 3, * *p* < 0.05, ** *p* < 0.01, *** *p* < 0.001).

To evaluate the protective effect of JAC4 on Rot-induced PD mice, we constructed a chronic Rot exposure mouse model (Figure 2A). At the end of the experiment, the disease model group mice showed significant weight loss compared with the normal control group (*p* < 0.01), while there was no significant difference in either of the JAC4 intervention groups (*p* > 0.05, Figure S1B). JAC4 is beneficial in preventing weight loss. To evaluate the efficacy of JAC4 in PD, we conducted behavioral tests of PD-like motor symptoms. In the open field test, a shorter travel distance was shown by the disease model mice in comparison with the normal control mice, and JAC4 prophylactic administration improved the mice’s spontaneous movement ability (Figure 2B,C), while no significant improvement was measured in the JAC4 therapeutic administration mice (*p* > 0.05, Figure S1C,D). Shorter times taken to move from the top to the bottom of the pole and longer latency periods to fall off the rotarod were observed in the JAC4 prophylactic administration mice (Figure 2D,E), but there was no effect on mice induced by JAC4 therapeutic intervention (Figure S1E,F). Then, we detected the expressions of TH and NLRP3 in the midbrain and striatum. The Western blot results showed that the upregulation of NLRP3 and downregulation of TH in the Rot disease model mice midbrain were reversed by JAC4 prophylactic administration (Figure 2F and Figure S2A), while these changes could not be detected in the JAC4 therapeutic mice (Figure S1G,H). Additionally, the same results could be obtained for the striatum using prophylactic administration (Figure 2F and Figure S2B). Moreover, our results showed that JAC4 prophylactic administration could reduce the pathological changes in alpha synuclein (α-SYN) in the midbrain (*p* < 0.05, Figure 2F and Figure S2B), but there was no statistical significance in the p-α-SYN (Ser129)/α-SYN ratio for the striatum (*p* > 0.05, Figure 2F and Figure S2D). Additionally, the Western blot results demonstrated that JAC4 prophylactic treatment could improve dopaminergic transmission (Figure S2E,F).

To further confirm the neuroprotective effect of JAC4 prophylactic administration, dopaminergic neurons in the substantia nigra pars compacta (SNc) were observed using immunofluorescent staining. We found that the loss of dopaminergic neurons was salvaged (Figure 2G,H). Hence, JAC4 prophylactic administration had an obvious neuroprotective effect on the Rot mouse model. It is known that the NLRP3 inflammasome may be involved in the pathogenesis of PD [20]. To determine the potential mechanism of JAC4 in anti-inflammation among Rot exposure mice, RNA was extracted from the mouse midbrain tissues, and the qRT-PCR results showed that the upregulation of NLRP3, caspase-1, IL-1β and IL-18 mRNA in the Rot-induced disease mice was reversed by JAC4 prophylactic intervention (Figure 2I–L). These results illustrate that prophylactic intervention using JAC4 could antagonize the Rot-induced activation of the NLRP3 inflammasome and multiple inflammation factors.

### 3.3. JAC4 Enhances Antioxidant Capacity and Attenuates the Activation of Astrocytes and Microglia in the Rotenone-Induced Mouse Model

**Figure 3:**
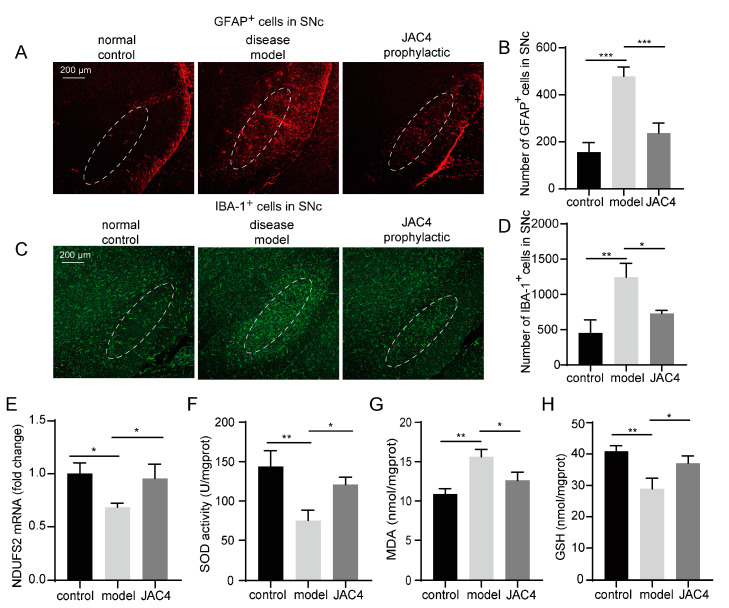
JAC4 enhances antioxidant capacity and attenuates the activation of astrocytes and microglia. (**A**–**D**) Immunofluorescence images and quantification of GFAP-positive (**A**,**B**) and IBA-1-positive (**C**,**D**) neurons in SNc. (**E**) The mRNA expression levels of the core subunit NDUFS2 of mitochondrial complex I in the midbrain. (**F**–**H**) The contents of SOD, MDA and GSH in the midbrain. The results are shown as the mean ± SEM (*n* = 3, * *p* < 0.05, ** *p* < 0.01, *** *p* < 0.001).

The proliferation and activation of astrocytes and microglia can be observed frequently in PD and always lead to the release of inflammatory factors. The GFAP-stained and IBA-1-stained images showed that Rot-exposure resulted in the activation of astrocytes and microglia in the SNc, while they were significantly blocked by JAC4 prophylaxis (*p* < 0.05, Figure 3A–D). Mitochondria are crucial for energy and Ros production [21]. Rot inhibits mitochondrial complex I activity, which is thought to be closely associated with PD [22]. We detected the mRNA expression levels of the core subunit NDUFS2 of mitochondrial complex I in the midbrain, and the results showed that JAC4 prophylactic improved the activity of mitochondrial complex I (Figure 3E). Given that the toxic effects of most pesticides are mainly related to oxidative stress events, we then examined the antioxidant status by measuring the contents of SOD, MDA and GSH in the midbrain tissues and sera. JAC4 preventive intervention reduced the overproduction of MDA induced by Rot and improved the activity of SOD and the production of GSH (Figure 3F–H and Figure S2G–I). Together, these results support the antioxidant and anti-inflammatory effects of JAC4 on Rot-induced disease mice.

### 3.4. JAC4 Alleviates Apoptosis and Inflammasome Formation In Vitro

**Figure 4:**
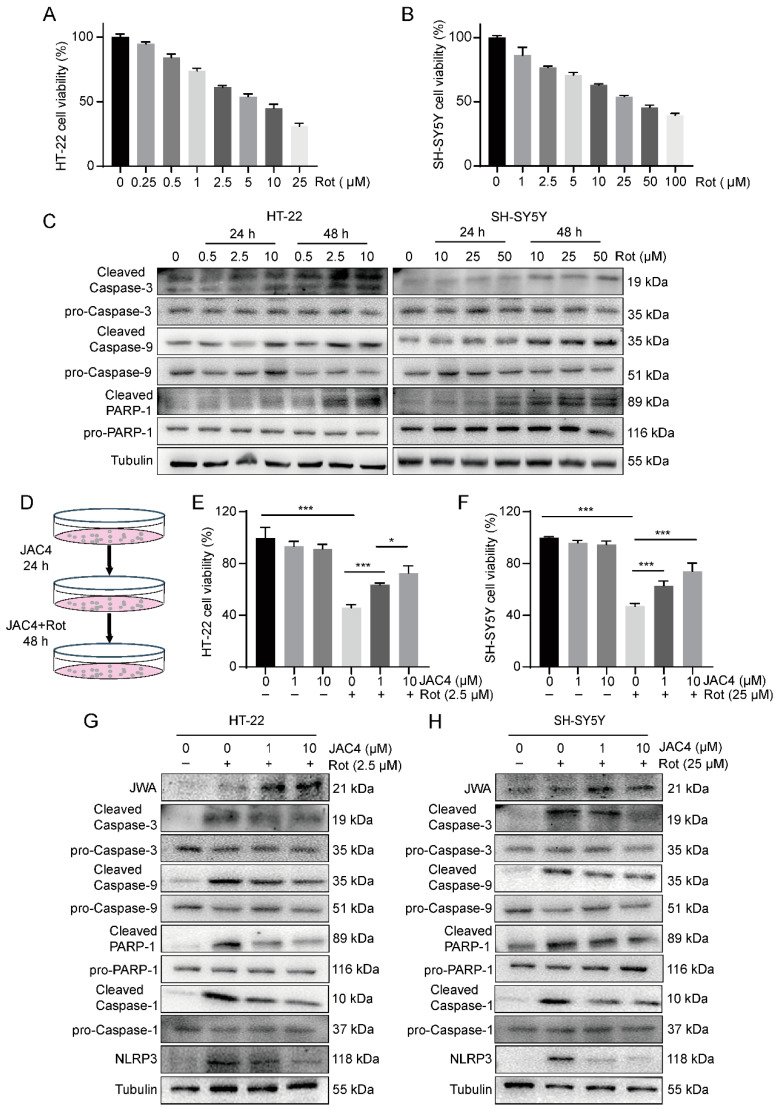
JAC4 alleviates cell apoptosis and inflammasome formation in vitro. (**A**,**B**) Cell viability at different doses Rot measured for 24 h in HT-22 (**A**) and SH-SY5Y cells (**B**). (**C**) The protein expression levels of caspase-3, caspase-9 and PARP-1 after 24 h and 48 h of Rot treatment at different doses. (**D**) The schematic diagram of the experimental design in vitro. Cells were pretreated with JAC4 for 24 h and co-treated with Rot for 48 h. (**E**,**F**) Cell viability of the TH-22 (**E**) and SH-SY5Y cells (**F**) was measured via CCK8 after treatment with different doses of JAC4 and Rot. (**G**,**H**) The protein expression levels of JWA, caspase-3, caspase-9, PARP-1, caspase-1 and NLRP3 in HT-22 (**G**) and SH-SY5Y (**H**). (*n* = 3, * *p* < 0.05, *** *p* < 0.001).

Next, we determined the protective effect of JAC4 in vitro by constructing Rot-induced models of HT-22 and SH-SY5Y cells to further verify its mechanisms. The results of the CCK8 assay demonstrated the toxicity of Rot, in a dose-dependent manner, towards the HT-22 and SH-SY5Y cells (Figure 4A,B). Moreover, the Western blot results showed (Figure 4C) that cleaved caspase-3, cleaved caspase-9 and cleaved PARP-1 were steadily increased after 48 h treatment with 2.5 µM Rot in the HT-22 cells (25 µM Rot in SH-SY5Y). Hence, we constructed models according to the above conditions and treated the cells with JAC4 at different concentrations (0, 1, 10 µM) to observe the effect of JAC4 in vitro (Figure 4D). The CCK8 results showed that JAC4 haf no toxic effects and could improve cell viability, which was suppressed by Rot (Figure 4E,F). As shown in the Western blot images displayed above (Figure 4G), Rot exposure elevated the levels of cleaved caspase-3, cleaved caspase-9 and cleaved PARP1, while JAC4 treatment reversed this damage in both cell lines. Similarly, JAC4 suppressed the upregulation of NLRP3 and active cleaved caspase-1 triggered by Rot.

### 3.5. JAC4 Suppresses Rotenone-Triggered Oxidative Stress and Mitochondrial Damage In Vitro

**Figure 5:**
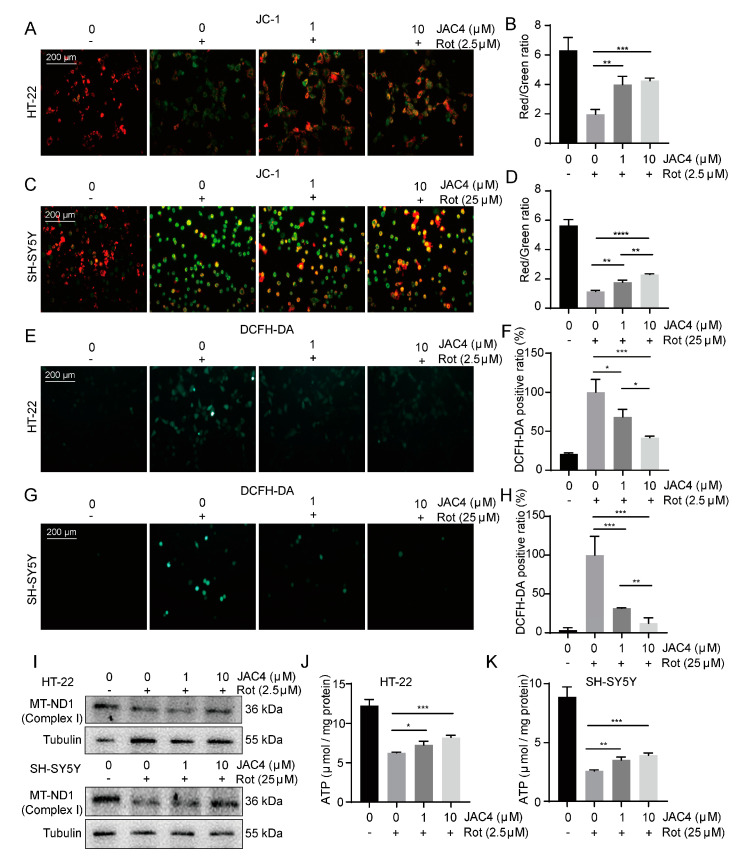
JAC4 alleviates rotenone-triggered oxidative stress and mitochondrial damage in vitro. (**A**–**D**) Mitochondrial membrane potential was detected using the JC-1 probe (**A**,**C**) and is shown as the JC-1 red/green ratio (**B**,**D**). (**E**–**H**) ROS accumulation in cells was detected via ROS assay (**E**,**G**) and analyzed using Image-J (**F**,**H**). (**I**) The protein expression levels of MT-ND1. (**J**,**K**) The contents of ATP in HT-22 (**J**) and SH-SY5Y (**K**). The results are shown as the mean ± SEM (*n* = 3, * *p* < 0.05, ** *p* < 0.01, *** *p* < 0.001), **** *p* < 0.0001).

Increasing evidence shows that the mechanisms of neurodegeneration in PD rely on oxidative reactions and mitochondrial dysfunction [23]. We measured mitochondrial membrane potential (ΔΨm) with JC-1 staining. The treatment of JAC4 protected cells from ΔΨm repression (Figure 5A–D). Moreover, as the results of the ROS assay demonstrated, ROS accumulation was significantly aggravated by Rot exposure in vitro (*p* < 0.05), while JAC4 promoted ROS scavenging (Figure 5E–H). Meanwhile, the mitochondrial complex I core subunit protein expression of MT-ND1 was decreased after Rot exposure and was prevented with JAC4 (Figure 5I). Moreover, JAC4 partly rescued Rot-induced ATP depletion (Figure 5J,K). The above results indicate that JAC4 ameliorates Rot-triggered oxidative stress and maintains the homeostasis of mitochondria.

### 3.6. JAC4 Inhibits Rot-Triggered NF-κB (p65) Nuclear Translocation

**Figure 6:**
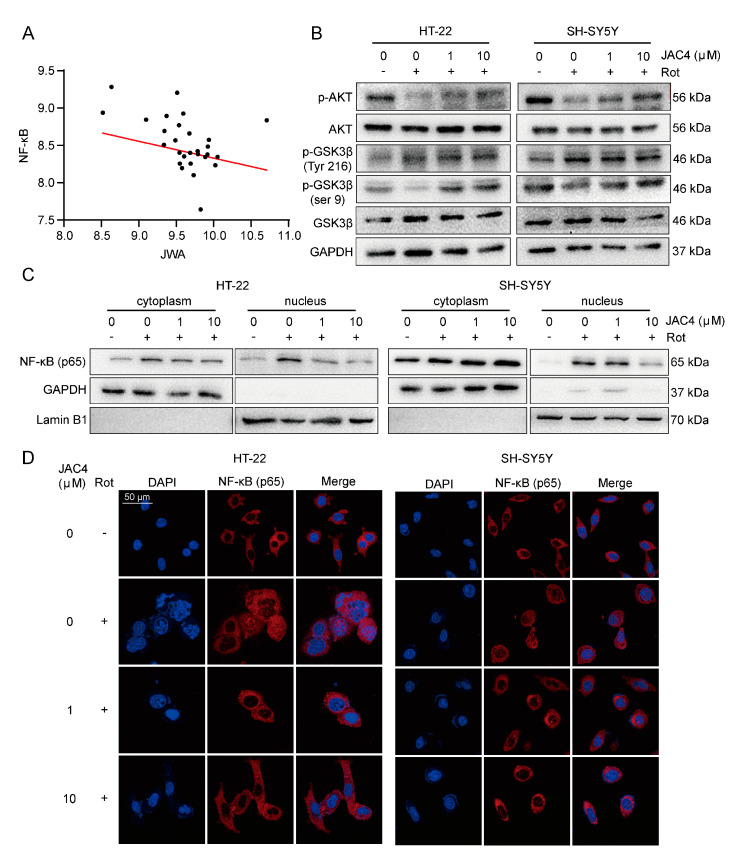
JAC4 inhibits NF-κB (p65) nuclear translocation. (**A**) Correlation analysis of the expressions of JWA and NF-κB in PD (GSE 6613, *n* = 50, r = −0.27). (**B**) The protein expression levels of p-AKT, AKT, p-GSK3β (Ser9), p-GSK3β (Tyr216) and GSK3β. (**C**) The protein expression levels of NF-κB in the nucleus and cytoplasm. (**D**) Immunofluorescences image of NF-κB location.

**Figure 7:**
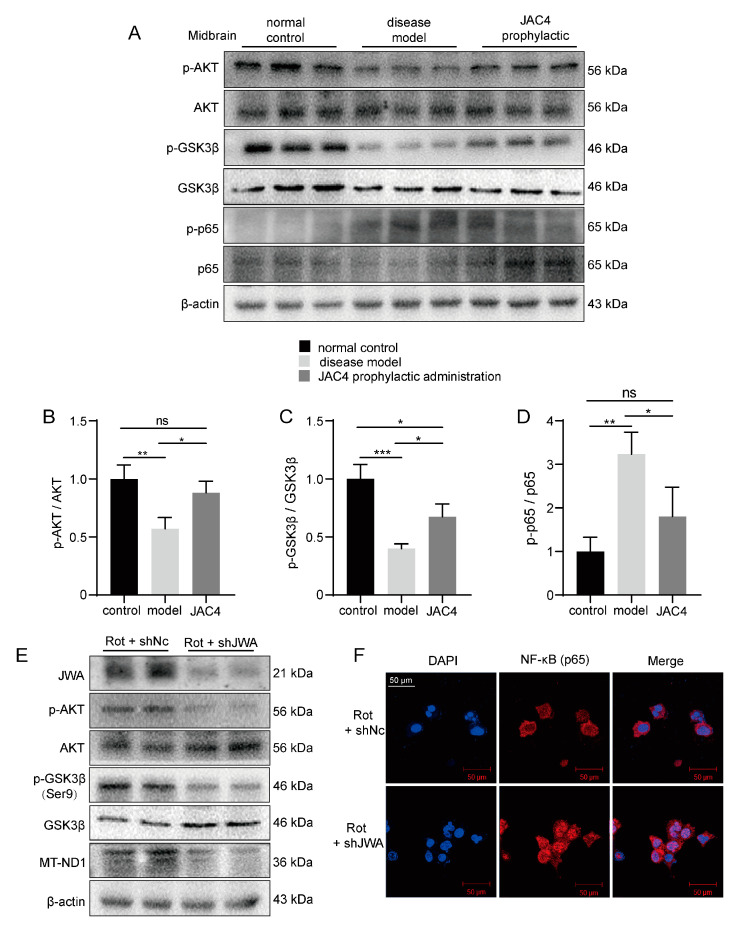
The verification of the mechanism in mice the midbrain and HT-22 cells. (**A**–**D**) The protein expression levels of p-AKT, AKT, p-GSK3β (Ser9), GSK3β, p-p65 and p65 in the midbrain of mice and their resulting quantized statistics. (**E**,**F**) The protein expression levels of JWA, p-AKT, AKT, p-GSK3β (Ser9), GSK3β and MT-ND1. (**E**) Immunofluorescence images of NF-κB location (**F**) in HT-22 cells Rot-treated for 48 h after shJWA transfection. The results are shown as the mean ± SEM (*n* = 3, * *p* < 0.05, ** *p* < 0.01, *** *p* < 0.001, ^ns^ *p* > 0.05).

NF-κB is a key transcriptional factor and considered necessary in the activation of NLRP3 inflammasome [12]. The public database analysis (GSE6613) showed a negative correlation between the expressions of JWA and NF-κB in the whole blood of PD patients (Figure 6A). Meanwhile, after separating the cytoplasm and nucleus, the Western blot results showed that Rot induced higher p65 expression in the nucleus, while JAC4 antagonized this nuclear translocation (Figure 6C). Additionally, the same results were obtained through the immunofluorescence staining of p65 (Figure 6D). How does JWA regulate p65? Evidence shows that AKT and GSK3β are abnormally expressed in PD, and AKT inhibits dopaminergic neuron apoptosis and oxidative stress by increasing the GSK3β phosphorylation of Ser9 and decreasing p65 phosphorylation [24,25]. Our results demonstrated that Rot exposure decreased AKT and GSK3β (Ser9) phosphorylation levels and increased GSK3β (Tyr216) phosphorylation levels, which activated oxidative stress and NF-κB signaling. However, JAC4 repressed these signaling by increasing p-AKT and p-GSK3β (Ser9) instead of decreasing p- GSK3β (Tyr216) (Figure 6B). Meanwhile, the same results were presented in the Rot-induced mouse model (Figure 7A–D). In addition, to further verify the above results, we constructed JWA knockdown cells via transfection with the shJWA plasmid. As exhibited in the Western blot analysis (Figure 7E), compared with the control transfected with the shNC plasmid, the phosphorylation levels of AKT, GSK3β (Ser9) and MT-ND1 (a mitochondrial complex I core subunit) further decreased and the nucleus translocation of p65 increased after JWA knockdown (Figure 7F). Based on the above evidence, our research supports the notion that JAC4 exerts a neuroprotective effect by repressing oxidative stress and neuroinflammation in the Rot-induced PD model via the JWA-mediated AKT/GSK3β/NF-κB signaling pathway.

## 4. Discussion

**Figure 8:**
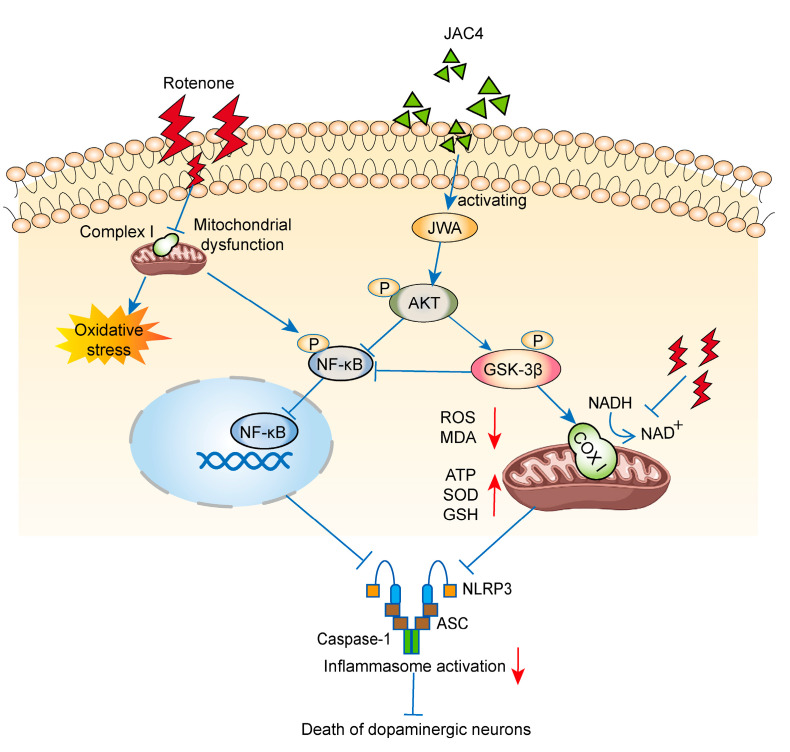
Schematic diagram of the molecular mechanism of the neuroprotective effect of JAC4 in the Rot-induced PD model.

There are currently no treatments that can stop or slow the progression of Parkinson’s disease. Although dopaminergic drugs or dopamine receptor agonists can be used to improve motor symptoms of PD, long-term treatment with these drugs often leads to drug resistance and serious complications such as impulse control disorders and can even worsen non-motor symptoms in patients [26,27]. Rotenone, a functional analogue of MPTP that is widely used in agriculture, inhibits complex I in the electron transport chain, which can lead to oxidative damage and progressive neurodegenerative diseases [28,29]. Rot-induced PD models are also widely recognized and used. This study focused on the neuroprotective effect and molecular mechanism of JAC4 in Rot-induced PD. For the first time, early JAC4 intervention was found to markedly improve Rot-induced motor dysfunction and dopaminergic neuron loss by inhibiting inflammasome activation, which relies on the maintenance of normal mitochondrial function and the reduction in NF-κB nuclear translocation (Figure 8).

Studies have recognized that mitochondrial dysfunction, oxidative stress and inflammatory change can lead to neuron dysfunction and death [30,31,32]. JWA is an active environmental response gene and is widely involved in cell responses to various stimulations, such as oxidative stress [33,34] and heat shock stress [35], and plays a role in antioxidants and the repair of DNA damage [15]. Previous studies have shown that the expression of JWA was downregulated in an MPTP-induced chronic PD model, and JWA knockout in astrocytes aggravated dopaminergic neuron loss [17]. JAC4 has been shown to reduce X-ray-induced intestine epithelium damage by repressing oxidative stress and enhancing DNA repair [14]. However, the effect and potential mechanism of JAC4 in PD remain unknown.

In this study, Rot-induced models were constructed in vivo and in vitro to investigate the protective effect. JAC4 intervention showed significant resistance to body weight loss in Rot-induced disease mice (*p* < 0.05). Moreover, our results showed that the prophylactic administration of JAC4 significantly improved motor functions and antagonized the progressive loss of dopaminergic neurons, while these improvements were not observed in the therapeutic administration group. This may be related to the fact that the death of neurons is irreversible. Research has shown that a large number of neurons (over 30%) are damaged when motor dysfunction occurs [28]. Therefore, JAC4 is an effective candidate agent for preventive or early intervention in Rot-induced PD. Mitochondria are important sources of ROS and perform pivotal roles in ATP production, calcium homeostasis and apoptosis [36,37]. A lower mtDNA content and more mtDNA mutations are always observed in the SN of PD patients [38,39,40]. By detecting mitochondrial complex I core subunit NDUFS2 mRNA or MT-ND1 protein expression, we found that JAC4 alleviated Rot-induced mitochondrial complex I damage. ATP is released during apoptosis and the release of intracellular ATP, and ATP intervention can act as a signal for NLRP3 activation [41]. In our study, JAC4 increased the ATP content and ΔΨm as a result of the suppression of mitochondrial damage. ROS reduction has been used as a treatment strategy for PD [42,43]. In this study, it was demonstrated that JAC4 improves mitochondrial damage and represses ROS production. 

The activation and proliferation of astrocytes and microglia can be observed in the substantia nigra of PD patients and usually lead to the release of cytokines and inflammatory chemokines [44,45]. The prophylactic administration of JAC4 mitigated the activation of astrocytes and microglia in the SNc, which is consistent with previous research [17,18,46]. NLRP3 inflammasome activation, assembled by NLRP3-ASC-caspase-1, may drive neurodegenerative diseases [47]. The NLRP3 inflammasome and NLRP3-dependent inflammatory cytokine release have been found in the blood of PD patients [11,48]. JAC4 reduced the expressions of NLRP3, caspase-1, IL-1β and IL-18, which supports the notion that JAC4 can antagonize the activation of the NLRP3 inflammasome induced by Rot. The activation of NLRP3 usually involves two steps: (1) NF-κB and other possible inflammatory transcription factors activate NLRP3, where the signal provided by the NF-κB activator is necessary for NLPR3 activation; and (2) external stimuli lead to the dysfunction of mitochondrial, lysosome and ion redistribution [49,50]. We found a negative correlation between the expression levels of JWA and NF-κB in the whole blood of PD patients. Meanwhile, JAC4 was shown to reduce NF-κB nuclear translocation by activating JWA to promote AKT phosphorylation in vivo and in vitro. Similarly, previous studies have demonstrated that JWA deficiency leads to the activation of the IKKβ-IκB-NF-κB signaling pathway [18].

GSK-3β, a multifunctional kinase, is involved in proinflammatory processes and apoptosis via the downstream PI3K/AKT pathway, and its inactivation can reduce the expression of p-α-SYN [51,52,53]. GSK-3β blocking has been shown to reduce oxidative damage in some neuronal models, and NF-κB activation is an important pro-inflammatory pathway promoted by GSK-3β [54,55]. Therefore, GSK-3β inactivation may be a key factor in inhibiting oxidative stress and inflammation in neurons. JAC4 inhibited the activity of GSK-3β by promoting the phosphorylation of Ser9, which alleviates oxidative stress. 

There are still some shortcomings of this study. Since JWA and TH showed the same trend, the question of whether JWA could be a marker for early diagnosis or intervention requires further study. The specific mechanism of JAC4 in antagonizing mitochondrial complex I damage still needs to be explored further. Additionally, it is not yet known whether JAC4 also plays a role in PD models without the inhibition of complex I. In addition, the intervention doses and initiation time required for JAC4 therapy to work need to be further optimized. Nevertheless, we demonstrated that JAC4, in part, could inhibit the activation of the NLRP3 inflammasome by inhibiting the AKT/GSK3β/NF-κB pathway and oxidative stress. These results provide proof that JAC4 could possibly serve as a novel, effective agent for PD treatment in the clinic.

## 5. Conclusions

This study demonstrated the neuroprotective effect of JAC4, a JWA gene activator, in Rot-induced PD. Early JAC4 intervention markedly improved motor dysfunction and dopaminergic neuron loss. Mechanistically, JAC4 prophylactic treatment reduced oxidative stress damage by reversing mitochondrial complex I damage, reducing NF-κB nuclear translocation and repressing NLRP3 inflammasome activation. Thus, our study suggests that the JWA gene activator compound JAC4 could serve as a novel, effective agent for PD prevention.

## Data Availability

All data are presented within the article and its additional files.

## Supplementary Materials

The following supporting information can be downloaded at: https://www.mdpi.com/article/10.3390/antiox12051134/s1, Figure S1: Effect of JAC4 therapeutic intervention in Rot-induced PD mice. (A) The chemical structural formula. (B) The body weight curves of the Rot exposure mouse model (n = 10). (C–F) Behavioral test results for the normal control, disease model and JAC4 therapeutic intervention mice. Movement track (C) and distance (D) of mice over 5 min in the open field. Climbing time from the top to the bottom of the pole in the pole test (E). Latency to fall within 5 min in the rotarod test (F). (G,H) The protein expression levels of TH, JWA and NLRP3 in the midbrain and the quantitative results. The results are shown as the mean ± SEM (* *p* < 0.05, ** *p* < 0.01, *** *p* < 0.001); Figure S2: (A,C) The quantitative results of the TH, NLRP3, and JWA protein levels in the midbrain (A) and striatum (C). (B,D) The quantitative results of the p-alpha synuclein (Ser129)/alpha synuclein ratio in the midbrain (B) and striatum (D). (E–F) The protein expression levels of dopamine transporter (DAT) in the striatum. (G–I) The contents of SOD, MDA and GSH in serum. The results are shown as the mean ± SEM (*n* = 3, * *p* < 0.05, ** *p* < 0.01, *** *p* < 0.001).
