# Supplementary material for: JAC4 Alleviates Rotenone-Induced Parkinson’s Disease through the Inactivation of the NLRP3 Signal Pathway
Source: Antioxidants (Basel). 2023 May 20;12(5):1134. doi: 10.3390/antiox12051134 (PMC10215424; doi:10.3390/antiox12051134)
Supplement: Supplementary file 1 [file antioxidants-12-01134-s001.zip › antioxidants-2322221-supplementary.pdf]

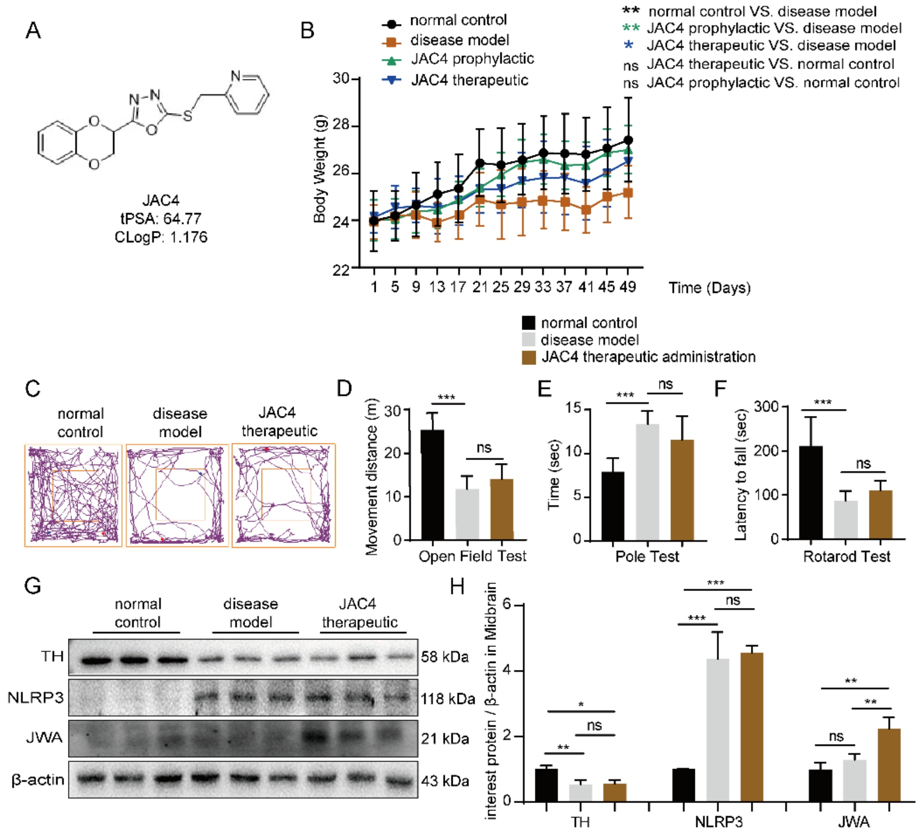

Figure S1: Effect of JAC4 therapeutic intervention in Rot-induced PD mice. (**A**) The chemical structural formula. (**B**) The body weight curves of Rot-exposure mouse model ($n = 10$). (**C–F**) Behavioral test results about normal control, disease model and JAC4 therapeutic intervention mice. Movement track (**C**) and distance (**D**) of mice during 5 min in the open field. Climbing time from the top of pole to the bottom in the pole test (**E**). Latency to fall within 5 min in the rotarod test (**F**). (**G, H**) The protein expression levels of TH, JWA and NLRP3 in midbrain and their quantitative results. The results are shown as the mean ± SEM (* $p < 0.05$, ** $p < 0.01$, *** $p < 0.001$, ^{ns} $p > 0.05$).

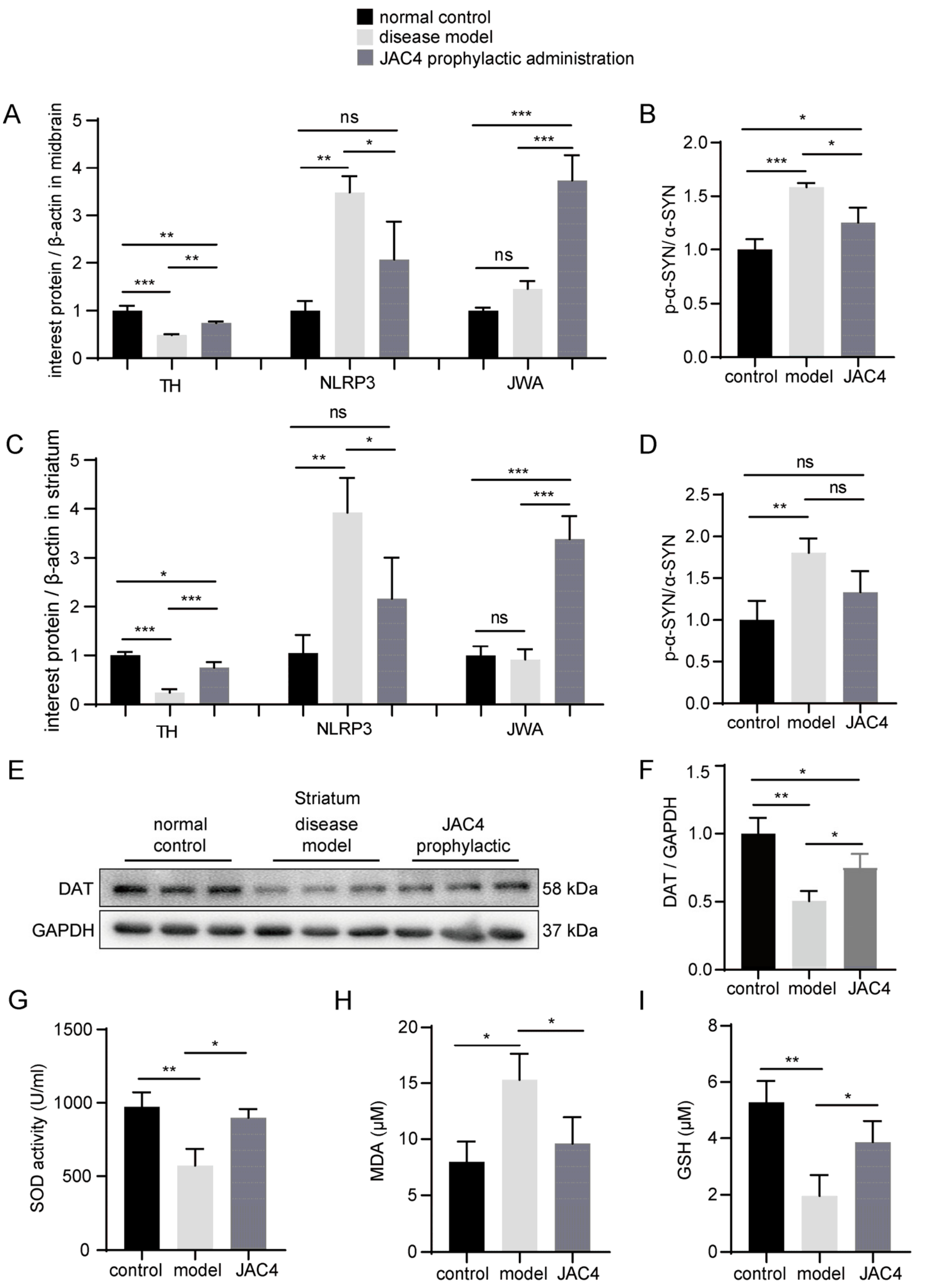

Figure S2: (**A**, **C**) The quantitative results of TH, NLRP3, and JWA protein levels in the midbrain (**A**) and striatum (**C**). (**B**, **D**) The quantitative results of p-alpha synuclein (Ser129)/ alpha synuclein ratio in the midbrain (**B**) and striatum (**D**). (**E**–**F**) The protein expression levels of dopamine transporter (DAT) in striatum. (**G**–**I**) The content of SOD, MDA and GSH in serum. The results are shown as the mean ± SEM ($n = 3$, * $p < 0.05$, ** $p < 0.01$, *** $p < 0.001$, ^{ns} $p > 0.05$).
